# A second monoclinic polymorph of *N*-(2,4-dinitro­phen­yl)-2,4-dinitro­aniline

**DOI:** 10.1107/S1600536812051288

**Published:** 2013-01-04

**Authors:** Yui Tokutome, Tsunehisa Okuno

**Affiliations:** a Department of Material Science and Chemistry, Wakayama University, Sakaedani, Wakayama, 640-8510, Japan

## Abstract

The title compound, C_12_H_7_N_5_O_8_, was previously described in space group *P*2_1_/*n* with *Z* = 4 [Wu *et al.* (2007 ▶). *Acta Cryst.* E**63**, o4194]. The current monoclinic *P*2_1_/*c* polymorph was obtained from a mixed solution of dichloro­methane and hexane. The dihedral angle between the benzene rings is 44.16 (5)°, smaller than in the previously reported polymorph [56.3 (2)°]. As a result of the steric hinderance of the nitro groups, hydrogen bonding is limited intramolecularly. The dihedral angles between the phenyl rings and their attached nitro groups are 18.97 (6) and 17.71 (5)° at the 2-position, and 18.52 (6) and 32.41 (6)° at the 4-position.

## Related literature

For the preparation of the title compound, see: Elliot & Smith (2000 ▶). For general background, see Espinoza & Thornton (1994 ▶); Farrell *et al.* (1985 ▶); Chattanathan & Kalidas (1971 ▶); Southgate & Hall (1971 ▶); Stewart & O’Donnell (1964 ▶). For the first monoclinic polymorph, see: Wu *et al.* (2007 ▶).
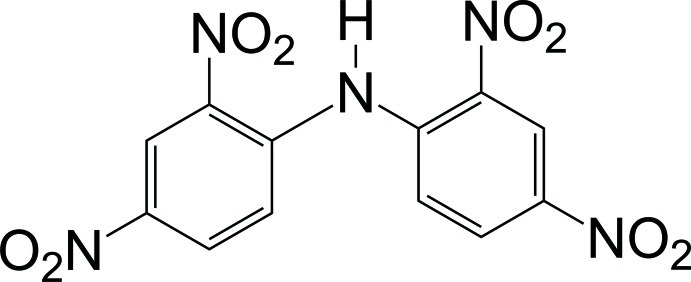


## Experimental

### 

#### Crystal data

C_12_H_7_N_5_O_8_
*M* *_r_* = 349.22
Monoclinic, 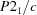

*a* = 12.827 (4) Å
*b* = 7.4997 (18) Å
*c* = 15.486 (4) Å
β = 113.906 (4)°
*V* = 1362.0 (6) Å^3^
*Z* = 4
Mo *K*α radiation
μ = 0.15 mm^−1^
*T* = 93 K
0.10 × 0.10 × 0.06 mm

#### Data collection

Rigaku Saturn724+ diffractometer
Absorption correction: numerical (*NUMABS*; Rigaku, 1999 ▶) *T*_min_ = 0.980, *T*_max_ = 0.991
10755 measured reflections
3119 independent reflections
2767 reflections with *F* ^2^ > 2σ(*F* ^2^)
*R*_int_ = 0.025

#### Refinement

*R*[*F* ^2^ > 2σ(*F* ^2^)] = 0.038
*wR*(*F* ^2^) = 0.105
*S* = 1.06
3119 reflections
230 parameters
1 restraint
H atoms treated by a mixture of independent and constrained refinement
Δρ_max_ = 0.30 e Å^−3^
Δρ_min_ = −0.24 e Å^−3^

### 

Data collection: *CrystalClear* (Rigaku, 2008 ▶); cell refinement: *CrystalClear*; data reduction: *CrystalClear*; program(s) used to solve structure: *SHELXD* (Schneider, *et al.*, 2002 ▶); program(s) used to refine structure: *SHELXL97* (Sheldrick, 2008 ▶); molecular graphics: *ORTEP-3* (Farrugia, 2012 ▶); software used to prepare material for publication: *CrystalStructure* (Rigaku, 2010 ▶).

## Supplementary Material

Crystal structure: contains datablock(s) global, I. DOI: 10.1107/S1600536812051288/ff2093sup1.cif

Structure factors: contains datablock(s) I. DOI: 10.1107/S1600536812051288/ff2093Isup2.hkl

Supplementary material file. DOI: 10.1107/S1600536812051288/ff2093Isup3.cml

Additional supplementary materials:  crystallographic information; 3D view; checkCIF report

## Figures and Tables

**Fig. 1.**
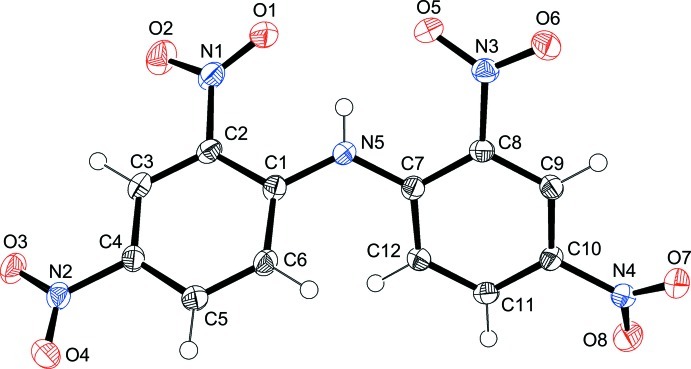
The asymmetric unit of the title compound with atom-numbering scheme. Displacement ellipsoids are drawn at the 50% probability level and H atoms are shown as small spheres.

**Fig. 2.**
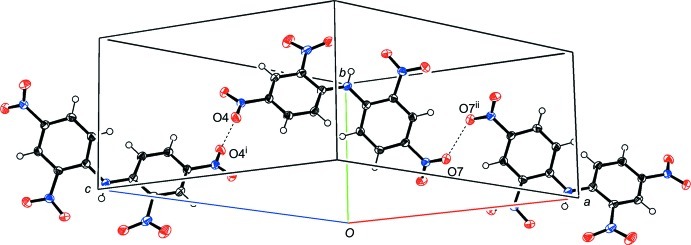
A view of the intermolecular interactions in the title compound. [Symmetry codes: (i) -x, -y + 1, -z + 1; (ii) -x + 1, -y + 1, -z.]

## supplementary crystallographic information

### Comment

The title compound, (I), is a derivative of nitrodiphenylamines which were used in nonlinear optical materials (Southgate & Hall, 1971). And the title compound was also used in smokeless gunpowder as a stabilizer (Espinoza & Thornton, 1994). Previously, (I) was isolated in a monoclinic *P*2_1_/*n* polymorph with *Z* = 4 (Wu *et al.*, 2007). A new monoclinic *P*2_1_/*c* polymorph was obtained by recrystallization from a mixed solution of dichloromethane and hexane.

The bond lengths and angles of the current molecule were almost similar to those of the reported one. However the significant difference was recognized at the dihedral angle between the two benzene rings. Although the angle of the reported molecule was 56.3 (2)°, that of the current molecule was 44.16 (5)°. Owing to the relatively small dihedral angle, the intramolecular distances between the N-bound H atom and the O atoms of the nitro groups at 2-position became close (Table 1). Because of the steric hinderance of the nitro groups, hydrogen-bondings are limited within the molecule.

The intermolecular contact was recognized between the oxygen atoms of the nitro groups, where the distances were 2.8032 (14) Å for O4···O4^i^ and 2.8859 (17) Å for O7···O7^ii^ [Symmetry codes: (i) -*x*, -*y* + 1, -*z* + 1; (ii) -*x* + 1, -*y* + 1, -*z*] (Figure 2).

### Experimental

Preparation of the title compound was carried out according to the reported procedure (Elliot & Smith, 2000). Single crystals with sufficient quality for X-ray crystallographical analysis were prepared by recrystallization from a mixed solution of dichloromethane and hexane.

### Refinement

The C-bound H atoms were placed at ideal positions and were refined as riding on their parent C atoms. *U*_iso_(H) values of the H atoms were set at 1.2*U*_eq_(parent atom). The N-bound H atom was obtained from a difference Fourier map and was refined isotropically with the restriction of N—H range between 0.807 Å and 0.847 Å.

### Crystal data

**Table:**

C_12_H_7_N_5_O_8_	*F*(000) = 712.00
*M**_r_* = 349.22	*D*_x_ = 1.703 Mg m^−^^3^
Monoclinic, *P*2_1_/*c*	Mo *K*α radiation, λ = 0.71075 Å
Hall symbol: -P 2ybc	Cell parameters from 3252 reflections
*a* = 12.827 (4) Å	θ = 1.7–25.0°
*b* = 7.4997 (18) Å	µ = 0.15 mm^−^^1^
*c* = 15.486 (4) Å	*T* = 93 K
β = 113.906 (4)°	Block, yellow
*V* = 1362.0 (6) Å^3^	0.10 × 0.10 × 0.06 mm
*Z* = 4	

### Data collection

**Table:**

Rigaku Saturn724+ diffractometer	2767 reflections with *F*^2^ > 2σ(*F*^2^)
Detector resolution: 7.111 pixels mm^-1^	*R*_int_ = 0.025
ω scans	θ_max_ = 27.5°
Absorption correction: numerical (*NUMABS*; Rigaku, 1999)	*h* = −16→16
*T*_min_ = 0.980, *T*_max_ = 0.991	*k* = −9→9
10755 measured reflections	*l* = −20→17
3119 independent reflections	

### Refinement

**Table:**

Refinement on *F*^2^	Secondary atom site location: difference Fourier map
*R*[*F*^2^ > 2σ(*F*^2^)] = 0.038	Hydrogen site location: inferred from neighbouring sites
*wR*(*F*^2^) = 0.105	H atoms treated by a mixture of independent and constrained refinement
*S* = 1.06	*w* = 1/[σ^2^(*F*_o_^2^) + (0.0621*P*)^2^ + 0.3959*P*] where *P* = (*F*_o_^2^ + 2*F*_c_^2^)/3
3119 reflections	(Δ/σ)_max_ < 0.001
230 parameters	Δρ_max_ = 0.30 e Å^−^^3^
1 restraint	Δρ_min_ = −0.24 e Å^−^^3^
Primary atom site location: structure-invariant direct methods	

### Special details

**Table:**

Refinement. Refinement was performed using all reflections. The weighted *R*-factor (*wR*) and goodness of fit (*S*) are based on *F*^2^. *R*-factor (gt) are based on *F*. The threshold expression of *F*^2^ > 2.0 σ(*F*^2^) is used only for calculating *R*-factor (gt).

### Fractional atomic coordinates and isotropic or equivalent isotropic displacement parameters (Å^2^)

**Table:**

	*x*	*y*	*z*	*U*_iso_*/*U*_eq_	
O1	0.20047 (11)	1.17542 (13)	0.26262 (8)	0.0365 (3)	
O2	0.05323 (10)	1.20201 (15)	0.29618 (8)	0.0394 (3)	
O3	−0.01586 (9)	0.79244 (13)	0.50055 (8)	0.0311 (3)	
O4	0.10386 (8)	0.57497 (13)	0.56109 (7)	0.0266 (3)	
O5	0.38633 (8)	1.06930 (12)	0.19345 (7)	0.0269 (3)	
O6	0.51942 (9)	0.95498 (13)	0.15832 (8)	0.0320 (3)	
O7	0.49908 (9)	0.34690 (13)	0.05612 (8)	0.0307 (3)	
O8	0.32685 (9)	0.24110 (14)	−0.00794 (8)	0.0342 (3)	
N1	0.13430 (11)	1.11698 (16)	0.29532 (8)	0.0275 (3)	
N2	0.06502 (9)	0.69826 (15)	0.50524 (8)	0.0226 (3)	
N3	0.43254 (9)	0.94107 (14)	0.17261 (8)	0.0219 (3)	
N4	0.39828 (10)	0.34574 (15)	0.04416 (8)	0.0239 (3)	
N5	0.27075 (10)	0.84682 (15)	0.25374 (8)	0.0214 (3)	
C1	0.22104 (10)	0.81023 (17)	0.31590 (9)	0.0203 (3)	
C2	0.15384 (11)	0.93838 (16)	0.33691 (9)	0.0208 (3)	
C3	0.10120 (10)	0.90156 (17)	0.39745 (9)	0.0213 (3)	
C4	0.11840 (10)	0.73735 (17)	0.43976 (9)	0.0204 (3)	
C5	0.18727 (11)	0.60873 (18)	0.42471 (9)	0.0224 (3)	
C6	0.23805 (11)	0.64557 (17)	0.36361 (9)	0.0225 (3)	
C7	0.29803 (10)	0.72531 (16)	0.19943 (8)	0.0187 (3)	
C8	0.37954 (10)	0.76573 (16)	0.16216 (8)	0.0188 (3)	
C9	0.41439 (10)	0.64030 (17)	0.11350 (9)	0.0195 (3)	
C10	0.36121 (11)	0.47701 (17)	0.09526 (9)	0.0205 (3)	
C11	0.27257 (11)	0.43627 (16)	0.12204 (9)	0.0206 (3)	
C12	0.24341 (11)	0.55830 (16)	0.17484 (9)	0.0203 (3)	
H1	0.2801 (16)	0.952 (2)	0.2451 (14)	0.038 (5)*	
H3	0.0544	0.9880	0.4093	0.0255*	
H5	0.1991	0.4970	0.4561	0.0268*	
H6	0.2855	0.5583	0.3534	0.0270*	
H9	0.4734	0.6666	0.0933	0.0234*	
H11	0.2329	0.3262	0.1042	0.0247*	
H12	0.1849	0.5293	0.1953	0.0243*	

### Atomic displacement parameters (Å^2^)

**Table:**

	*U*^11^	*U*^22^	*U*^33^	*U*^12^	*U*^13^	*U*^23^
O1	0.0599 (8)	0.0210 (5)	0.0403 (6)	0.0029 (5)	0.0325 (6)	0.0031 (5)
O2	0.0495 (7)	0.0330 (6)	0.0393 (6)	0.0225 (5)	0.0216 (6)	0.0098 (5)
O3	0.0316 (6)	0.0280 (6)	0.0434 (6)	−0.0010 (4)	0.0251 (5)	−0.0082 (5)
O4	0.0290 (5)	0.0291 (6)	0.0226 (5)	−0.0063 (4)	0.0114 (4)	−0.0003 (4)
O5	0.0327 (5)	0.0179 (5)	0.0320 (6)	−0.0010 (4)	0.0151 (5)	−0.0030 (4)
O6	0.0302 (5)	0.0300 (6)	0.0426 (6)	−0.0109 (4)	0.0216 (5)	−0.0085 (5)
O7	0.0326 (6)	0.0273 (6)	0.0407 (6)	0.0021 (4)	0.0235 (5)	−0.0013 (5)
O8	0.0425 (6)	0.0285 (6)	0.0356 (6)	−0.0094 (5)	0.0200 (5)	−0.0136 (5)
N1	0.0387 (7)	0.0217 (6)	0.0225 (6)	0.0071 (5)	0.0128 (5)	0.0006 (5)
N2	0.0233 (6)	0.0234 (6)	0.0239 (6)	−0.0053 (5)	0.0124 (5)	−0.0069 (5)
N3	0.0248 (6)	0.0201 (6)	0.0208 (5)	−0.0039 (4)	0.0092 (5)	−0.0016 (4)
N4	0.0316 (6)	0.0194 (6)	0.0253 (6)	−0.0016 (5)	0.0164 (5)	−0.0010 (5)
N5	0.0291 (6)	0.0163 (6)	0.0223 (6)	0.0017 (5)	0.0140 (5)	−0.0001 (4)
C1	0.0214 (6)	0.0210 (6)	0.0189 (6)	0.0018 (5)	0.0087 (5)	−0.0010 (5)
C2	0.0238 (6)	0.0174 (6)	0.0198 (6)	0.0030 (5)	0.0074 (5)	−0.0005 (5)
C3	0.0209 (6)	0.0208 (6)	0.0219 (6)	0.0028 (5)	0.0084 (5)	−0.0045 (5)
C4	0.0209 (6)	0.0223 (6)	0.0201 (6)	−0.0015 (5)	0.0104 (5)	−0.0032 (5)
C5	0.0251 (6)	0.0197 (6)	0.0234 (6)	0.0034 (5)	0.0110 (5)	0.0024 (5)
C6	0.0258 (6)	0.0205 (7)	0.0238 (6)	0.0066 (5)	0.0125 (5)	0.0018 (5)
C7	0.0207 (6)	0.0185 (6)	0.0169 (6)	0.0027 (5)	0.0076 (5)	0.0013 (5)
C8	0.0208 (6)	0.0166 (6)	0.0186 (6)	−0.0010 (5)	0.0076 (5)	0.0004 (5)
C9	0.0202 (6)	0.0204 (6)	0.0196 (6)	−0.0003 (5)	0.0097 (5)	0.0010 (5)
C10	0.0241 (6)	0.0184 (6)	0.0207 (6)	0.0011 (5)	0.0108 (5)	−0.0018 (5)
C11	0.0228 (6)	0.0164 (6)	0.0224 (6)	−0.0008 (5)	0.0091 (5)	0.0005 (5)
C12	0.0218 (6)	0.0190 (6)	0.0218 (6)	0.0007 (5)	0.0107 (5)	0.0019 (5)

### Geometric parameters (Å, º)

**Table:**

O1—N1	1.232 (3)	C3—C4	1.3702 (19)
O2—N1	1.225 (2)	C4—C5	1.390 (2)
O3—N2	1.2327 (17)	C5—C6	1.377 (3)
O4—N2	1.2256 (15)	C7—C8	1.417 (2)
O5—N3	1.2391 (16)	C7—C12	1.4101 (17)
O6—N3	1.2254 (19)	C8—C9	1.388 (2)
O7—N4	1.2297 (18)	C9—C10	1.3743 (18)
O8—N4	1.2277 (15)	C10—C11	1.393 (3)
N1—C2	1.4632 (18)	C11—C12	1.376 (2)
N2—C4	1.465 (3)	N5—H1	0.818 (16)
N3—C8	1.4589 (17)	C3—H3	0.950
N4—C10	1.458 (2)	C5—H5	0.950
N5—C1	1.381 (3)	C6—H6	0.950
N5—C7	1.3780 (19)	C9—H9	0.950
C1—C2	1.414 (2)	C11—H11	0.950
C1—C6	1.4099 (19)	C12—H12	0.950
C2—C3	1.387 (3)		
			
O1···O5	3.088 (2)	C11···O3^xii^	3.2472 (17)
O1···N5	2.6468 (17)	C11···O5^ix^	3.1014 (16)
O1···C1	2.8417 (18)	C11···O6^viii^	3.3694 (16)
O1···C3	3.515 (2)	C11···C3^i^	3.4945 (18)
O2···C1	3.5815 (19)	C12···O1^ix^	3.3172 (19)
O2···C3	2.6708 (18)	C12···O3^i^	3.5105 (16)
O3···C3	2.723 (3)	C12···O4^i^	3.3640 (16)
O3···C5	3.542 (3)	C12···O6^viii^	3.1876 (16)
O4···C3	3.5146 (19)	C12···N2^i^	3.2553 (16)
O4···C5	2.739 (3)	O1···H1	2.035 (18)
O5···N5	2.6411 (18)	O2···H3	2.3713
O5···C7	2.8341 (17)	O3···H3	2.4482
O5···C9	3.5178 (18)	O4···H5	2.4671
O6···C7	3.592 (2)	O5···H1	2.04 (3)
O6···C9	2.6662 (17)	O6···H9	2.3573
O7···C9	2.7557 (19)	O7···H9	2.5185
O7···C11	3.512 (3)	O8···H11	2.5626
O8···C9	3.4712 (18)	N1···H1	2.61 (3)
O8···C11	2.795 (2)	N1···H3	2.5624
N1···N5	2.915 (2)	N2···H3	2.6046
N3···N5	2.915 (2)	N2···H5	2.6223
C1···C4	2.785 (3)	N3···H1	2.62 (3)
C1···C12	2.989 (2)	N3···H9	2.5577
C2···C5	2.7699 (19)	N4···H9	2.5895
C3···C6	2.791 (2)	N4···H11	2.6420
C6···C7	3.000 (3)	N5···H6	2.6197
C6···C12	3.024 (3)	N5···H12	2.6261
C7···C10	2.790 (2)	C1···H3	3.3077
C8···C11	2.7718 (18)	C1···H5	3.2878
C9···C12	2.789 (3)	C1···H12	2.7283
O1···O4^i^	3.4158 (17)	C2···H1	2.55 (3)
O1···O8^ii^	3.3131 (18)	C2···H6	3.2704
O1···C11^iii^	3.325 (2)	C3···H5	3.2688
O1···C12^iii^	3.3172 (19)	C4···H6	3.2423
O2···O3^iv^	3.371 (2)	C5···H3	3.2742
O2···C1^v^	3.3316 (18)	C5···H12	3.5909
O2···C2^v^	3.1637 (17)	C6···H1	3.12 (2)
O2···C3^v^	3.2158 (17)	C6···H12	2.5655
O2···C4^v^	3.4275 (18)	C7···H6	2.7548
O2···C6^v^	3.5735 (18)	C7···H9	3.3097
O3···O2^iv^	3.371 (2)	C7···H11	3.2916
O3···O3^iv^	3.1407 (16)	C8···H1	2.56 (3)
O3···O4^vi^	2.9841 (15)	C8···H12	3.2676
O3···C3^iv^	3.219 (2)	C9···H11	3.2733
O3···C11^v^	3.2472 (17)	C10···H12	3.2409
O3···C12^ii^	3.5105 (16)	C11···H9	3.2788
O4···O1^ii^	3.4158 (17)	C12···H1	3.117 (16)
O4···O3^vi^	2.9841 (15)	C12···H6	2.5934
O4···O4^vi^	2.8032 (14)	H1···H6	3.3837
O4···O5^ii^	3.5329 (15)	H1···H12	3.3738
O4···N2^vi^	2.8537 (16)	H5···H6	2.3240
O4···N5^ii^	2.9413 (15)	H6···H12	2.2687
O4···C7^ii^	2.9516 (15)	H11···H12	2.3223
O4···C8^ii^	3.4491 (17)	O1···H6^iii^	3.1886
O4···C12^ii^	3.3640 (16)	O1···H11^iii^	2.8814
O5···O4^i^	3.5329 (15)	O1···H12^iii^	2.8286
O5···O6^vii^	3.5844 (15)	O2···H5^iii^	3.2826
O5···O8^iii^	3.1686 (18)	O2···H12^v^	3.3700
O5···N4^iii^	3.1550 (18)	O3···H3^iv^	2.3380
O5···C8^vii^	3.2783 (15)	O3···H5^vi^	3.4591
O5···C9^vii^	3.0922 (15)	O3···H11^v^	2.6054
O5···C10^iii^	3.3717 (18)	O3···H12^v^	3.4239
O5···C11^iii^	3.1014 (16)	O3···H12^ii^	3.3497
O6···O5^viii^	3.5844 (15)	O4···H1^ii^	2.837 (17)
O6···O7^iii^	3.2981 (16)	O4···H11^xiii^	3.3680
O6···O8^iii^	3.4901 (15)	O4···H12^ii^	3.5296
O6···N4^iii^	3.4499 (16)	O5···H5^i^	3.5166
O6···C6^vii^	3.563 (3)	O5···H9^vii^	3.1398
O6···C7^vii^	3.2040 (15)	O5···H11^iii^	2.7001
O6···C8^vii^	3.4525 (17)	O6···H6^vii^	2.6946
O6···C9^vii^	3.565 (2)	O6···H12^vii^	3.5867
O6···C10^vii^	3.493 (2)	O7···H1^viii^	3.325 (16)
O6···C11^vii^	3.3694 (16)	O7···H6^viii^	3.3385
O6···C12^vii^	3.1876 (16)	O7···H9^x^	2.4801
O7···O6^ix^	3.2981 (16)	O8···H5^xi^	2.3330
O7···O7^x^	2.8859 (17)	O8···H6^xi^	3.0019
O7···N4^x^	3.3340 (19)	O8···H9^x^	3.3962
O7···N5^viii^	3.2158 (15)	N2···H3^iv^	3.3587
O7···C1^viii^	3.3356 (17)	N2···H12^ii^	3.3961
O7···C6^viii^	3.4348 (19)	N3···H5^i^	3.5048
O7···C9^x^	3.241 (3)	N4···H5^xi^	3.4883
O8···O1^i^	3.3131 (18)	N4···H9^x^	3.1761
O8···O5^ix^	3.1686 (18)	C3···H11^ii^	3.5899
O8···O6^ix^	3.4901 (15)	C3···H12^v^	3.4898
O8···N1^i^	3.2275 (16)	C8···H5^i^	3.5662
O8···N3^ix^	3.4127 (17)	C11···H3^i^	3.3966
O8···C2^i^	3.4864 (16)	C12···H3^xii^	3.5408
O8···C5^xi^	3.1086 (18)	H1···O4^i^	2.837 (17)
O8···C6^xi^	3.4391 (18)	H1···O7^vii^	3.325 (16)
N1···O8^ii^	3.2275 (16)	H1···H9^vii^	3.5260
N2···O4^vi^	2.8537 (16)	H1···H11^iii^	3.4536
N2···N2^vi^	3.3812 (18)	H3···O3^iv^	2.3380
N2···C7^ii^	3.3198 (15)	H3···N2^iv^	3.3587
N2···C12^ii^	3.2553 (16)	H3···C11^ii^	3.3966
N3···O8^iii^	3.4127 (17)	H3···C12^v^	3.5408
N3···N4^iii^	3.5570 (18)	H3···H11^ii^	3.2700
N3···C9^vii^	3.4334 (17)	H3···H12^v^	2.8487
N3···C10^vii^	3.5052 (17)	H5···O2^ix^	3.2826
N4···O5^ix^	3.1550 (18)	H5···O3^vi^	3.4591
N4···O6^ix^	3.4499 (16)	H5···O5^ii^	3.5166
N4···O7^x^	3.3340 (19)	H5···O8^xiii^	2.3330
N4···N3^ix^	3.5570 (18)	H5···N3^ii^	3.5048
N5···O4^i^	2.9413 (15)	H5···N4^xiii^	3.4883
N5···O7^vii^	3.2158 (15)	H5···C8^ii^	3.5662
C1···O2^xii^	3.3316 (18)	H5···H11^xiii^	3.2421
C1···O7^vii^	3.3356 (17)	H6···O1^ix^	3.1886
C2···O2^xii^	3.1637 (17)	H6···O6^viii^	2.6946
C2···O8^ii^	3.4864 (16)	H6···O7^vii^	3.3385
C3···O2^xii^	3.2158 (17)	H6···O8^xiii^	3.0019
C3···O3^iv^	3.219 (2)	H9···O5^viii^	3.1398
C3···C11^ii^	3.4945 (18)	H9···O7^x^	2.4801
C4···O2^xii^	3.4275 (18)	H9···O8^x^	3.3962
C5···O8^xiii^	3.1086 (18)	H9···N4^x^	3.1761
C6···O2^xii^	3.5735 (18)	H9···H1^viii^	3.5260
C6···O6^viii^	3.563 (3)	H11···O1^ix^	2.8814
C6···O7^vii^	3.4348 (19)	H11···O3^xii^	2.6054
C6···O8^xiii^	3.4391 (18)	H11···O4^xi^	3.3680
C7···O4^i^	2.9516 (15)	H11···O5^ix^	2.7001
C7···O6^viii^	3.2040 (15)	H11···C3^i^	3.5899
C7···N2^i^	3.3198 (15)	H11···H1^ix^	3.4536
C8···O4^i^	3.4491 (17)	H11···H3^i^	3.2700
C8···O5^viii^	3.2783 (15)	H11···H5^xi^	3.2421
C8···O6^viii^	3.4525 (17)	H12···O1^ix^	2.8286
C9···O5^viii^	3.0922 (15)	H12···O2^xii^	3.3700
C9···O6^viii^	3.565 (2)	H12···O3^xii^	3.4239
C9···O7^x^	3.241 (3)	H12···O3^i^	3.3497
C9···N3^viii^	3.4334 (17)	H12···O4^i^	3.5296
C10···O5^ix^	3.3717 (18)	H12···O6^viii^	3.5867
C10···O6^viii^	3.493 (2)	H12···N2^i^	3.3961
C10···N3^viii^	3.5052 (17)	H12···C3^xii^	3.4898
C11···O1^ix^	3.325 (2)	H12···H3^xii^	2.8487
			
O1—N1—O2	123.29 (13)	N5—C7—C12	122.07 (14)
O1—N1—C2	118.88 (13)	C8—C7—C12	116.61 (13)
O2—N1—C2	117.82 (15)	N3—C8—C7	122.37 (12)
O3—N2—O4	124.45 (15)	N3—C8—C9	115.66 (13)
O3—N2—C4	117.57 (12)	C7—C8—C9	121.97 (12)
O4—N2—C4	117.98 (12)	C8—C9—C10	118.25 (14)
O5—N3—O6	123.16 (11)	N4—C10—C9	117.99 (14)
O5—N3—C8	118.54 (13)	N4—C10—C11	119.90 (12)
O6—N3—C8	118.28 (12)	C9—C10—C11	122.10 (14)
O7—N4—O8	124.24 (14)	C10—C11—C12	118.83 (12)
O7—N4—C10	117.88 (11)	C7—C12—C11	121.75 (15)
O8—N4—C10	117.87 (13)	C1—N5—H1	116.4 (17)
C1—N5—C7	126.68 (12)	C7—N5—H1	116.7 (17)
N5—C1—C2	121.48 (12)	C2—C3—H3	120.863
N5—C1—C6	121.49 (13)	C4—C3—H3	120.846
C2—C1—C6	117.00 (14)	C4—C5—H5	120.467
N1—C2—C1	122.18 (15)	C6—C5—H5	120.467
N1—C2—C3	115.87 (13)	C1—C6—H6	119.323
C1—C2—C3	121.95 (12)	C5—C6—H6	119.339
C2—C3—C4	118.29 (13)	C8—C9—H9	120.873
N2—C4—C3	118.75 (13)	C10—C9—H9	120.874
N2—C4—C5	118.97 (12)	C10—C11—H11	120.582
C3—C4—C5	122.25 (15)	C12—C11—H11	120.588
C4—C5—C6	119.07 (13)	C7—C12—H12	119.124
C1—C6—C5	121.34 (14)	C11—C12—H12	119.126
N5—C7—C8	121.27 (12)		
			
O1—N1—C2—C1	−20.15 (16)	C2—C1—C6—C5	2.79 (16)
O1—N1—C2—C3	160.61 (10)	C6—C1—C2—N1	177.17 (9)
O2—N1—C2—C1	160.91 (10)	C6—C1—C2—C3	−3.63 (15)
O2—N1—C2—C3	−18.34 (15)	N1—C2—C3—C4	−178.79 (9)
O3—N2—C4—C3	19.08 (15)	C1—C2—C3—C4	1.96 (16)
O3—N2—C4—C5	−162.72 (9)	C2—C3—C4—N2	178.83 (9)
O4—N2—C4—C3	−160.66 (9)	C2—C3—C4—C5	0.69 (16)
O4—N2—C4—C5	17.55 (15)	N2—C4—C5—C6	−179.63 (9)
O5—N3—C8—C7	−17.98 (15)	C3—C4—C5—C6	−1.49 (16)
O5—N3—C8—C9	162.22 (9)	C4—C5—C6—C1	−0.34 (16)
O6—N3—C8—C7	163.56 (10)	N5—C7—C8—N3	−4.81 (15)
O6—N3—C8—C9	−16.23 (14)	N5—C7—C8—C9	174.97 (9)
O7—N4—C10—C9	32.16 (16)	N5—C7—C12—C11	−178.78 (9)
O7—N4—C10—C11	−149.31 (11)	C8—C7—C12—C11	3.78 (15)
O8—N4—C10—C9	−147.92 (11)	C12—C7—C8—N3	172.65 (9)
O8—N4—C10—C11	30.62 (16)	C12—C7—C8—C9	−7.57 (14)
C1—N5—C7—C8	−159.52 (10)	N3—C8—C9—C10	−175.18 (8)
C1—N5—C7—C12	23.16 (16)	C7—C8—C9—C10	5.02 (15)
C7—N5—C1—C2	−152.59 (10)	C8—C9—C10—N4	−179.96 (9)
C7—N5—C1—C6	29.51 (16)	C8—C9—C10—C11	1.54 (16)
N5—C1—C2—N1	−0.82 (16)	N4—C10—C11—C12	176.35 (9)
N5—C1—C2—C3	178.38 (9)	C9—C10—C11—C12	−5.18 (17)
N5—C1—C6—C5	−179.23 (9)	C10—C11—C12—C7	2.33 (16)

Symmetry codes: (i) *x*, −*y*+3/2, *z*−1/2; (ii) *x*, −*y*+3/2, *z*+1/2; (iii) *x*, *y*+1, *z*; (iv) −*x*, −*y*+2, −*z*+1; (v) −*x*, *y*+1/2, −*z*+1/2; (vi) −*x*, −*y*+1, −*z*+1; (vii) −*x*+1, *y*+1/2, −*z*+1/2; (viii) −*x*+1, *y*−1/2, −*z*+1/2; (ix) *x*, *y*−1, *z*; (x) −*x*+1, −*y*+1, −*z*; (xi) *x*, −*y*+1/2, *z*−1/2; (xii) −*x*, *y*−1/2, −*z*+1/2; (xiii) *x*, −*y*+1/2, *z*+1/2.

### Hydrogen-bond geometry (Å, º)

**Table:**

*D*—H···*A*	*D*—H	H···*A*	*D*···*A*	*D*—H···*A*
N5—H1···O1	0.818 (16)	2.035 (16)	2.6468 (17)	131 (3)
N5—H1···O5	0.818 (16)	2.038 (16)	2.6411 (18)	130 (2)
N5—H1···N1	0.818 (16)	2.609 (16)	2.915 (2)	103.8 (19)
N5—H1···N3	0.818 (16)	2.622 (16)	2.915 (2)	102.9 (18)

## References

[bb1] Chattanathan, N. & Kalidas, C. (1971). *Aust. J. Chem.* **24**, 83–88.

[bb2] Elliot, M. S. & Smith, F. J. (2000). *Propell. Explos. Pyrot.* **25**, 31–36.

[bb3] Espinoza, E. & Thornton, J. (1994). *Anal. Chim. Acta*, **288**, 57–69.

[bb4] Farrell, P. G., Terrier, F. & Schaal, R. (1985). *Tetrahedron Lett.* **26**, 2435–2438.

[bb5] Farrugia, L. J. (2012). *J. Appl. Cryst.* **45**, 849–854.

[bb6] Rigaku (1999). *NUMABS* Rigaku Corporation, Tokyo, Japan.

[bb7] Rigaku (2008). *CrystalClear* Rigaku Corporation, Tokyo, Japan.

[bb8] Rigaku (2010). *CrystalStructure* Rigaku Corporation, Tokyo, Japan.

[bb9] Schneider, T. R. & Sheldrick, G. M. (2002). *Acta Cryst.* D**58**, 1772–1779. 10.1107/s0907444902011678 12351820

[bb10] Sheldrick, G. M. (2008). *Acta Cryst.* A**64**, 112–122. 10.1107/S0108767307043930 18156677

[bb11] Southgate, P. D. & Hall, D. S. (1971). *Appl. Phys. Lett.* **18**, 456–459.

[bb12] Stewart, R. & O’Donnell, J. P. (1964). *Can. J. Chem.* **42**, 1694–1698.

[bb13] Wu, D.-L., Jia, Z.-L., Shi, J.-P. & Lu, G.-Y. (2007). *Acta Cryst.* E**63**, o4194.

